# Supplementary material for: Content-rich biological network constructed by mining PubMed abstracts
Source: BMC Bioinformatics. 2004 Oct 8;5:147. doi: 10.1186/1471-2105-5-147 (PMC528731; doi:10.1186/1471-2105-5-147)
Supplement: Additional File 2 — The original results of the above study (non-essential files are deleted to keep the file size under the limit set by BMC bioinformatics). [file 1471-2105-5-147-S2.bz2 › chilibotAdditionalFile2/dip30/104ID9362454E434/html/BCL2L1_BAK1.html]

 


 **BCL2L1** and **BAK1** 
  
Found 265 abstracts in PubMed, retrieved 30.  
 

 What does Google say? 
 PDF only 
| .edu only 

---

**Interactive relationship** (e.g. stimulation, inhibition, etc)

**Inhibitory relationship**- RESULTS Butyrate induced a clear shift of the mitochondrial bcl rheostat towards a proapoptotic constellation, as demonstrated by upregulation of proapoptotic bak  [ **BAK1** ]  accompanied by reduced antiapoptotic bcl x  [ **BCL2L1** ]  L levels.  Ref: 12477768 Gut, 2003
**Stimulatory relationship**- Unlike Bcl 2, Bak  [ **BAK1** ]  stimulates several apoptosis pathways, and by interacting with Bcl 2 or Bcl XL  [ **BCL2L1** ] , it counteracts the anti cell death effect.  Ref: 12836337 Nucleic Acids Res Suppl, 2001
**Neutral relationship**- gossypol induced apoptosis triggers down regulation of anti apoptosis Bcl 2 members Bcl X  [ **BCL2L1** ]  L, Bag 1 and Mcl 1, up regulation of pro apoptosis Bcl 2 member Bak  [ **BAK1** ] , activation of caspase,, and 9, up regulation of Apaf 1, release of cytochrome c cyto c from mitochondria, and activation of both DFF45 and PARP.  Ref: 12818369 Biochem Pharmacol, 2003

**Non-interactive relationship** (e.g. studied together, co-existance, homology, etc.)

- The expression of the pro apoptotic members Bax, Bak  [ **BAK1** ] , Bid, and Bcl x  [ **BCL2L1** ]  was significantly decreased after TPN.  Ref: 12471468 Pediatr Surg Int, 2002
- Nonetheless, there were no significant alterations in the level of Bcl 2, Bcl X  [ **BCL2L1** ]  L, Bax and Bak  [ **BAK1** ]  by the proteasome inhibitor, nor any evidence of cytochrome cyt c release into cytosol from dying cells, suggesting that cyt c is not involved.  Ref: 12560102 FEBS Lett, 2003
- However, loss of Bax, Bak  [ **BAK1** ]  and Bcl Xs  [ **BCL2L1** ]  did not compromise sensitivity to TRAIL.  Ref: 12604406 Haematologica, 2003
- They can be pro apoptotic e.g. Bid, Bax, and Bak  [ **BAK1** ]  or anti apoptotic e.g. Bcl 2 and Bcl x  [ **BCL2L1** ]  L.  Ref: 12624108 J Biol Chem, 2003
- Furthermore, Bcl 2 family members Bad, Bak  [ **BAK1** ] , and Bcl xS  [ **BCL2L1** ]  protein levels were increased in FHIT transfected clones when compared with Panc 1 cells.  Ref: 12890991 Pancreatology, 2003
- this dominant negative activity requires both a failure of interaction with Bax and Bak  [ **BAK1** ]  through the BH1 domain of Bcl 2 and retention of the ability to interact with Bcl 2 and Bcl xL  [ **BCL2L1** ] .  Ref: 12644466 J Biol Chem, 2003
- PURPOSEWe hypothesized that apoptosis is a downstream event in erectile dysfunction, and pro apoptotic Bak  [ **BAK1** ]  and Bax and anti apoptotic Bcl 2 and Bcl x  [ **BCL2L1** ]  factors are involved in the etiology of diabetes induced erectile dysfunction.  Ref: 12796708 J Urol< MedlineTA>J Urol, 2003
- iv TGFbeta1 increased the levels of the anti apoptotic Bcl 2 and Bcl xL  [ **BCL2L1** ]  proteins, while having no significant effects on the pro apoptotic proteins Bax and Bak  [ **BAK1** ] , suggesting the activation of a transient survival mechanism activated in stromal cells as a parallel rescue response to the apoptosis inducing FasL protein.  Ref: 12569178 Mol Hum Reprod, 2003
- No difference in bak  [ **BAK1** ] , fas, bcl 2 or bcl x  [ **BCL2L1** ]  expression was observed among the groups.  Ref: 12756564 Virchows Arch< MedlineTA>Virchows Arch, 2003
- LPA had no effect on Bcl xl  [ **BCL2L1** ] , Bad, and Bak  [ **BAK1** ]  mRNA or protein expression.  Ref: 12684213 Am J Physiol Gastrointest Liver Physiol, 2003
- These two mutants still retained the ability to interact with wild type Bcl 2 and Bcl xL  [ **BCL2L1** ] , and abrogated the inhibitory effect of wild type Bcl 2 or Bcl xL  [ **BCL2L1** ]  on Bax or Bak  [ **BAK1** ]  induced apoptosis.  Ref: 12644466 J Biol Chem, 2003
- HSpin1 bound to Bcl 2 and apoptosis regulator Bcl X  [ **BCL2L1** ]  Bcl x  [ **BCL2L1** ]  L, but not to proapoptotic members such as Bcl 2 associated X protein and Bcl 2 homologous antagonist killer  [ **BAK1** ] , in cells treated with TNF alpha.  Ref: 12815463 Cell Death Differ, 2003
- Western blot analysis was performed with anti ERK1 2, Mcl 1, Bak  [ **BAK1** ] , Bcl x  [ **BCL2L1** ]  and Bax antibodies.  Ref: 12606824 Mol Med, 2002
- This increased apoptosis was accompanied by decreased antiapoptotic bcl 2 mRNA expression among bcl 2 related genes bcl 2, bax, bak  [ **BAK1** ] , mcl 1, and bcl X  [ **BCL2L1** ]  L S, and the effect was also more prominent in the cagA strains.  Ref: 12540563 Infect Immun, 2003
- We also analyzed the relationship between TCF 4 gene splicing isoforms, proliferation proliferating cell nuclear antigen labeling index, and apoptosis antiapoptotic factors Bcl 2 and Bcl x  [ **BCL2L1** ]  L, proapoptotic factors Bak  [ **BAK1** ]  and Bax, and caspase 3 in RCC samples.  Ref: 12796377 Clin Cancer Res< MedlineTA>Clin Cancer Res, 2003
- The objective was to evaluate the immunohistochemical expression of p53, p21, bax, bak  [ **BAK1** ] , fas, bcl 2 and bcl x  [ **BCL2L1** ]  proteins in 10 endometriomas, 20 benign ovarian tumours 10 mucinous, 10 serous and 30 malignant ovarian tumours 9 mucinous, 19 serous.  Ref: 12756564 Virchows Arch< MedlineTA>Virchows Arch, 2003
- These findings suggest that S. aureus activated PMC extend PMN life span by modulating Bcl xL  [ **BCL2L1** ]  and Bak  [ **BAK1** ]  gene expression and active caspases activity during acute inflammation and empyema.  Ref: 12645855 J Clin Immunol, 2003
